# Evaluation of Antimicrobial and Anticancer Activities of Selected Medicinal Plants of Himalayas, Pakistan

**DOI:** 10.3390/plants11010048

**Published:** 2021-12-24

**Authors:** Farzana Kausar, Kyung-Hwan Kim, Hafiz Muhammad Umer Farooqi, Muhammad Awais Farooqi, Muhammad Kaleem, Rooma Waqar, Atif Ali Khan Khalil, Fazli Khuda, Chethikkattuveli Salih Abdul Rahim, Kinam Hyun, Kyung-Hyun Choi, Abdul Samad Mumtaz

**Affiliations:** 1 Department of Plant Sciences, Quaid-i-Azam University, Islamabad 45320, Pakistan; kausaufarzana4915@gmail.com (F.K.); mkaleem@bs.qau.edu.pk (M.K.); rooma.waqar667@gmail.com (R.W.); 2 Department of Mechatronics Engineering, Jeju National University, Jeju-si 63243, Korea; rudghks624@gmail.com (K.-H.K.); umerfarooqi@jejunu.ac.kr (H.M.U.F.); awaisfarooqi@stu.jejunu.ac.kr (M.A.F.); abdul.rahim350@gmail.com (C.S.A.R.); gusrlska8204@jejunu.ac.kr (K.H.); 3 National Control Laboratory for Biologicals, Drug Regulatory Authority of Pakistan, Islamabad 44090, Pakistan; 4 Department of Biological Sciences, National University of Medical Sciences, Rawalpindi 46000, Pakistan; atif.ali@numspak.edu.pk; 5 Department of Pharmacy, University of Peshawar, Peshawar 25120, Pakistan; fazlikhuda@uop.edu.pk; 6 BioSpero, Inc., Jeju-si 63243, Korea

**Keywords:** *Prunus cornuta*, *Quercus semicarpifolia*, antibacterial, antifungal, anti-cancer

## Abstract

Medicinal plants are known for their diverse use in the traditional medicine of the Himalayan region of Pakistan. The present study is designed to investigate the anticancer and antimicrobial activities of *Prunus cornuta* and *Quercus* *semicarpifolia.* The anticancer activity was performed using cancerous human cell lines (HepG2, Caco-2, A549, MDA-MB-231, and NCI-H1437 carcinoma cells), while the antimicrobial activity was conducted with the agar-well diffusion method. Furthermore, toxicity studies were performed on alveolar and renal primary epithelial cells. Initially, different extracts were prepared by maceration techniques using *n*-hexane, chloroform, ethyl acetate, butanol, and methanol. The preliminary phytochemical screening showed the presence of secondary metabolites such as alkaloids, tannins, saponins, flavonoids, glycosides, and quinones. The chloroform extract of *P. cornuta* (PCC) exhibited significant inhibitory activity against *Acinetobacter baumannii* (16 mm) and *Salmonella enterica* (14.5 mm). The *A. baumannii* and *S. enterica* strains appeared highly susceptible to *n*-hexane extract of *P. cornuta* (PCN) with an antibacterial effect of 15 mm and 15.5 mm, respectively. The results also showed that the methanolic extracts of *Quercus semecarpifolia* (QSM) exhibited considerable antibacterial inhibitory activity in *A. baumannii* (18 mm), *Escherichia coli* (15 mm). The QSN and QSE extracts also showed good inhibition in *A. baumannii* with a 16 mm zone of inhibition. The *Rhizopus oryzae* strain has shown remarkable mycelial inhibition by PCM and QSN with 16 mm and 21 mm inhibition, respectively. Furthermore, the extracts of *P. cornuta* and *Q. semicarpifolia* exhibited prominent growth inhibition of breast (MDA-MB-231) and lung (A549) carcinoma cells with 19–30% and 22–39% cell viabilities, respectively. The gut cell line survival was also significantly inhibited by *Q. semicarpifolia* (24–34%). The findings of this study provide valuable information for the future development of new antibacterial and anticancer medicinal agents from *P. cornuta* and *Q. semicarpifolia* extracts.

## 1. Introduction

The use of wild medicinal plants to treat human ailments has been known since ancient times. For pharmacological purposes, the unveiling of the potential of natural sources like plants is not a new approach [1]. Nearly 80% of the world population in developing countries relies on plants to treat many ailments like infections, pain management, wound healing, reproductive problems, skin infections, gut issues, etc. [2]. Due to the adverse effects of chemical entities, the preference for herbal products over synthetic medicine increases day by day. Still, many studies are required to explore the potential use of indigenous plants for human illnesses such as cancer and infectious diseases [3].

Bacterial infections are considered to be a significant health problem due to the genetic modification of microbes against a selected drug, resulting in various globally resistant bacterial species [4]. Research to find a better substance from a natural source to overcome this health hazard is always in progress. Several plants have been investigated for antibacterial activities [5]. In addition to this, cancer incidence is one of the leading causes of death in developing and developed countries. Its increasing prevalence results in vast and continuous economic losses throughout the world. Adverse effects of chemotherapy on the human body, like nausea, vomiting, alopecia, etc., demand the search for novel candidate plant species or medicinal agents with less toxic effects on normal cells and more toxicity against cancerous cells [6]. Plants and their derivatives can be helpful in cancer therapy. However, some wild medicinal plants that are still obscured in their pharmacological potential have been scientifically evaluated [7].

**Figure 1:**
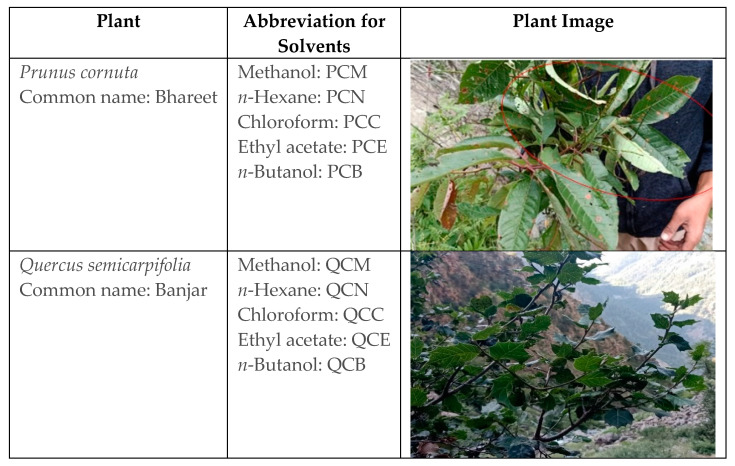
Details of plant species, common names, and solvents used for extraction.

*Prunus cornuta* Wall. ex Royle (Rosaceae) and *Quercus semicarpifolia* Sm (Fagaceae) are widely found in the Himalayan regions of Pakistan and India [8,9,10]. These plants contain numerous phytochemicals such as alkaloids, glycosides, flavonoids, and tannins. Traditionally, *P. cornuta* has been used to cure anemia. In contrast, *Q. semicarpifolia* is used to treat various ailments such as muscular pain, bleeding, chronic diarrhea, wound healing, inflammation, and dysentery [11,12,13]. Therefore, the present study reports the phytochemical composition and therapeutic validation of *P. cornuta* (PC) and *Q. semicarpifolia* (QS) plants, particularly with antimicrobial and anticancer effects (Figure 1).

## 2. Results

The present study evaluated the plant material’s in vitro antimicrobial activity and cytotoxic activity of butanoic, chloroform, methanolic, and n-hexane extracts against cancerous and normal human cell lines. First, qualitative phytochemical tests were performed to detect phytochemicals in the methanolic extracts. The antibacterial activity was then characterized using five different bacterial strains through the agar-well method for preliminary assessment of bacterial growth inhibition. 

### 2.1. Phytochemical Screening

The results of the phytochemical investigation of methanolic extracts are summarized in Table 1.

### 2.2. Antimicrobial Potential

#### 2.2.1. Antibacterial Effect

In this study, two strains, *A. baumannii* and *S. enterica*, were more sensitive than the other tested bacterial strains. Extracts showed the highest inhibition against *A. baumannii,* followed by *S. enterica.* Furthermore, extracts exhibited moderate activity against *B. subtilis*, *K. pneumoniae,* and *E. coli.* In PC extracts, the highest activity was observed by PCN and PCC (Table 2). The current study validated the excellent antibacterial activity of QS extract against *K. pneumoniae*, *E. coli*, *B. subtilis*, *S. enterica*, and *A. baumannii*, and QS extracts showed maximum inhibition with methanolic solvents, as shown in Table 2. All extracts exhibited potential bacterial inhibition activity from 9 to 18 mm to control (12 to 16 mm). In addition, both plant extracts showed significant antibacterial activity against *A. baumannii* as shown in Supplementary Data Figures S1 and S2.

#### 2.2.2. Antifungal Effect

*P. cornuta* and *Q. semicarpifolia* have shown no significant inhibition of the fungal isolates *A. flavus*, *A. niger,* and *Pythium sp*., but not for *R. oryzae*. The susceptibility of *R. oryzae* by *P. cornuta* was observed in the PCM and PCN extracts only. In the case of *Q. semicarpifolia,* the maximum mycelial inhibition was observed in QSE (21 mm), followed by PCM and PCN (16.6 mm), as shown in Table 3. The percentage of mycelial growth inhibition was significant in *R. oryzae,* followed by *F. fujikuroi* isolates. *R. oryzae* appeared susceptible to PCM and PCE extracts with 67 and 64% mycelial inhibition, respectively. On the other hand, *Q. Semicarpifolia* restricted the *R. oryzae* fungal growth up to 57% with QSM and QSB extracts as shown in Supplementary Data Figure S3. The maximum mycelial inhibition against *F. fujikuroi* pathogen was observed with PCC extract (59%) and QSE (54%) (Table 4).

### 2.3. Anticancer Activity

**Figure 2:**
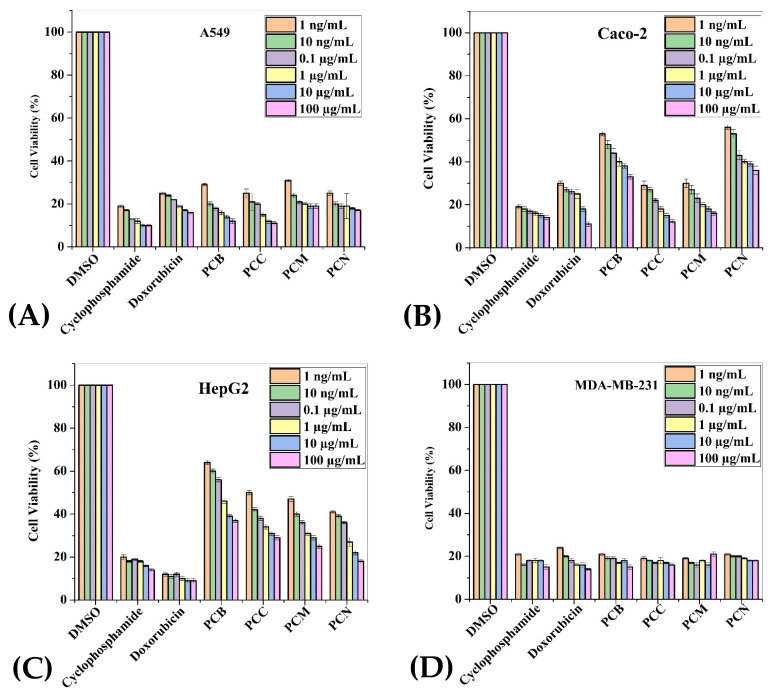

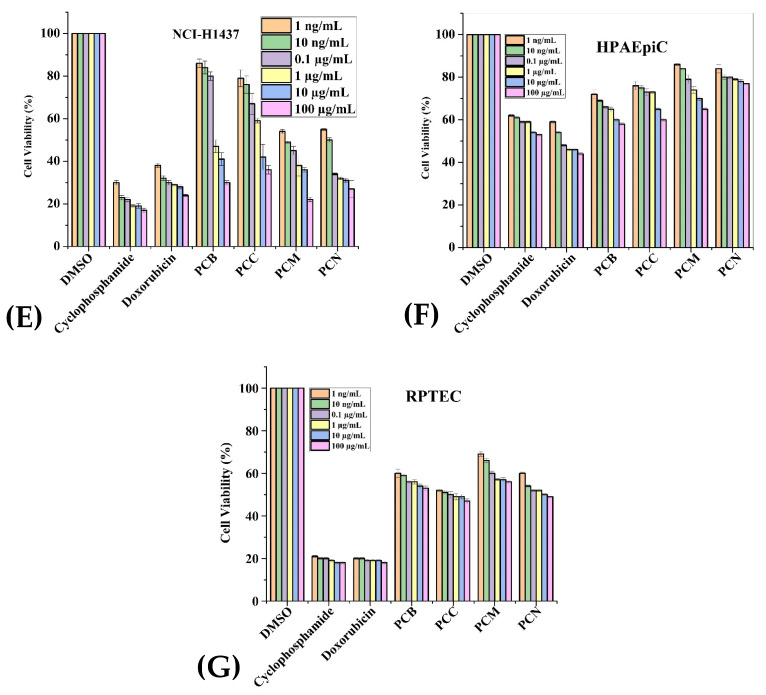
Cell viability: MTS assay histograms represent the percentage viability with respect to control cells (positive control: 30–40% viable cells) after exposure to: 1 ng/mL, 10 ng/mL, 0.1 µg/mL, 1 µg/mL, 10 µg/mL, 100 µg/mL of PCB, PCC, PCM, PCN extracts in A549 cells (**A**), Caco-2 (**B**), HepG2 (**C**), MDA-MB-231 (**D**), NCI-H1437 (**E**) cancerous cell lines and HPAEpiC (**F**) and RPTEC (**G**) cell lines. Data shown as mean ± SE (*n* = 3).

**Figure 3:**
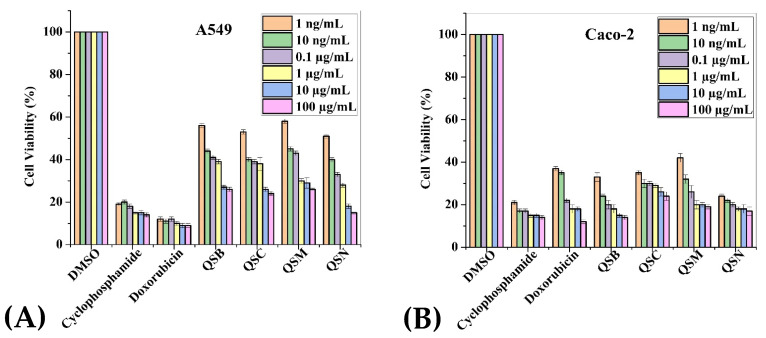

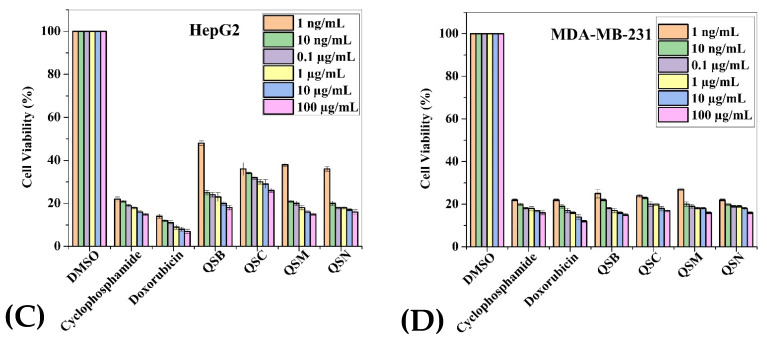

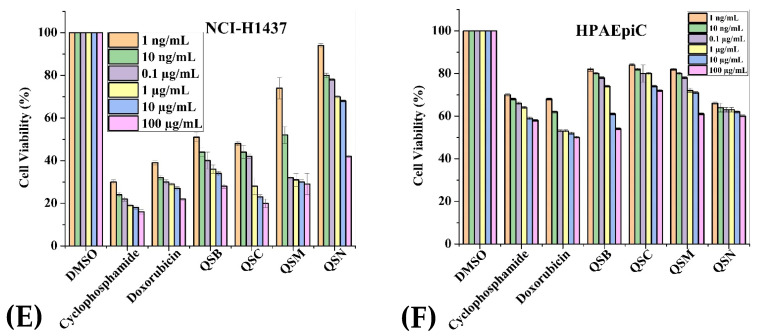

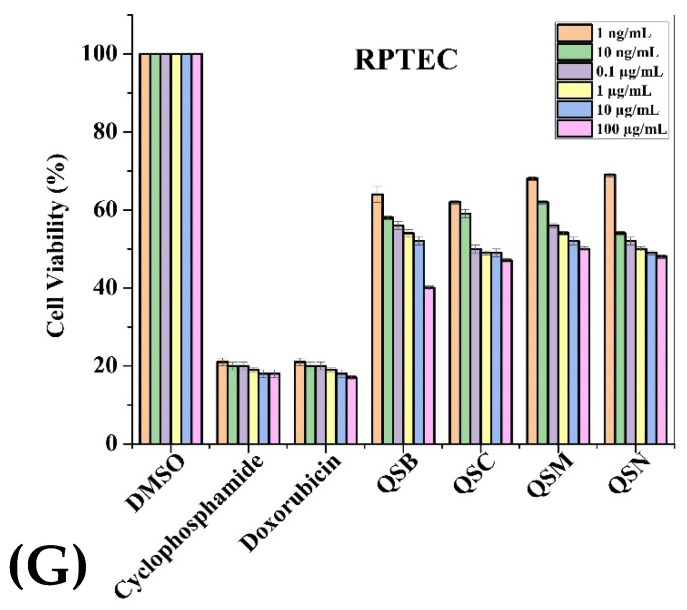
Cell viability: MTS assay histograms represent the percentage cell viability with respect to control cells (positive control: 20–30% viable cells) after exposure to: 1 ng/mL, 10 ng/mL, 0.1 µg/mL, 1 µg/mL, 10 µg/mL, 100 µg/mL of QSB, QSC, QSM, QSN extracts in A549 cells (**A**), Caco-2 (**B**), HepG2 (**C**), MDA-MB-231 (**D**), NCI-H1437 (**E**) cancerous cell lines and HPAEpiC (**F**) and RPTEC (**G**) cell lines. Data shown as mean ± SE (*n* = 3).

To assess the cytotoxic effect of *P. cornuta* and *Q. semicarpifolia* extracts on lung (A549), gut (Caco-2), liver (HepG2), breast (MDA-MB-231), and lung (NCI-H1437) cancer cell lines, an MTS assay was performed. The lower percentage cell viability values indicated a higher rate of cytotoxicity. Furthermore, the growth inhibition of cancerous cells was dose-dependent, where maximum growth inhibition was observed at the highest concentrations, i.e., 100 µg/mL (Figure 2 and 3). 

The inhibitory effect of *P. cornuta* extracts was highest against MD-MBA-231 and potent against A549 and Caco-2 cells (100 µg/mL) (Figure 2A,B,D, respectively). Moreover, PC crude extracts showed moderate activity against HepG2 and NCI-H1437 (Figure 2C,E, respectively). However, all extracts showed less inhibition of cell proliferation in NCI-HI437 cells and good inhibition in MDA-MB-231 (18–30%) compared with the standard drugs (17 to 27% cell viability). Further, the percentage cell viability rate was 54 to 76% in primary epithelial cells HPAEpiC and HRPTEpiC, providing safety data for this study, Figure 2F,G. In addition to this, extracts in different solvents showed a slightly different inhibition pattern against a specific type of cancerous cells lines. Chloroform extracts of *P. cornuta* showed the highest cytotoxic effect in Caco-2, A549, and MDA-MB-231 cancerous cells, signifying the antibacterial activity results. These findings also indicated that statistically significant (*p* = 0.001) growth inhibition had been observed against A549 and MDA-MB321 (Figure 2A,D). The percentage of cell viability by *P. cornuta* extracts is shown in Table 5.

The effect of *Q. semicarpifolia* extracts on the cell viability of breast and gut cell lines was 30–35% viability after treatment (Figure 3B,D), whereas the lung and liver cell lines had 35–69% cell viability in the order of A549 > HepG2 cells > NCI-H1437 (Figure 3A,C,E respectively, Table 5). In contrast, no significant effect of *Q. semicarpifolia* extracts was observed on normal cell lines (Figure 3F,G). However, positive control (doxorubicin, cyclophosphamide) inhibited cancer cell line growth with 17–27% cell viability. Furthermore, butanolic and *n*-hexane extracts in the QS plant exhibited low cell viability, providing remarkable retardation of cancerous cell proliferation. Thus, the results suggest that the *Q. semicarpifolia* extracts exhibit strong anti-proliferative ability without affecting the normal cells, as shown in Figure 3.

**Table 5:** Average (*n* = 3) % age cell viability of plant extracts against human-derived cancerous cell lines and healthy cell lines.

Extracts (100 µg/mL)	Cancerous Cell Lines					Normal Cell Lines	
	Caco-2	A549	HepG2	MDA-MB-231	NCI-H1437	HPAEpiC	HRPTEpiC
PCB	42.6	26.85	50.3	19	67.71	64.85	58.85
PCC	20.5	25.42	37.3	18	58.14	72.28	52.28
PCM	22.3	29.14	34.6	26.57	47.71	78.85	63.28
PCN	44.5	22	30.5	19.71	39.14	80.71	53.28
QSB	29.71	38.8	35.71	29.28	46.42	71.14	56
QSC	30.42	36.6	33	22.42	35.85	75.71	54.85
QSM	34.42	38.8	31.85	28.57	48.85	76.14	61.85
QSN	24	30.8	23.85	20.28	68.71	64.14	54
Doxo.	20.8	20.50	10.85	19	27.42	49.57	19.57
Cyclopho	17.14	14.42	17.85	18	20.71	57.14	18.71

## 3. Discussion

The Plant kingdom provides valuable and structurally diverse secondary metabolites to cure various human ailments. Several active compounds have been extracted and isolated to confirm the traditional uses of plants. Hence, there is growing interest in validating the biological potential of unusual plants, which further facilitates herbal medicine research [14]. The medicinal properties of *Prunus* and *Quercus* species are reported in various studies, including as diuretic, anti-inflammatory, antipyretic and refrigerant, astringent, heart disease protection, antibacterial hepatoprotective, antidiabetic, anticancer, gastroprotective, antioxidant, and cytotoxic activities [15,16]. In our study, the *P. cornuta* extracts exhibited potential antibacterial effect against all tested bacterial strains tested with an 11.5 mm minimum zone of inhibition. The results were similar to another study of antibacterial activity of *Prunus* species regarding *E. coli*, *K. pneumoniae,* and *B. subtilis* [15]. The similar diameter of zones of inhibition against *k. pneumoniae* were also observed by *P. domestica* (13 mm) [17]. The results also revealed that the *Q. semicarpifolia* extracts followed a similar trend of antibacterial activity, except for the inhibition in *S. enterica* (7–10 mm). A literature survey by Ema burlacu et al. also reported the microbial growth inhibition by various *Quercus* species against *K. pneumoniae*, *S. aureus*, and *A. baumannii* [18]. Thus confirming that the presence of phenolics, flavonoids, terpenoids, and tannins in *Quercus* species resulted in good antibacterial activity [19]. The occurrence of tannins is considered responsible for the antibacterial activity of the *Q. semicarpifolia* plant. A study by Sharba et al. observed the same inhibition pattern in *Q. infectoria* against *E. coli*, *S. aureus, C. albicans,* and other *Quercus* species [19,20].

Various anticancer drugs have been developed throughout time, but nowadays, resistance and side effects of cancer treatments demand new, highly efficient anticancer substances with low toxic effects [21]. The MTS assay method was applied, which quantified the viable cells after treating them with extracts of different concentrations. It involves the reduction of MTS tetrazolium dye to a soluble formazan dye by test cells. This reduction can be estimated by measuring the absorbance of the cells at 490–500 nm. Plant extracts were observed on different cancerous and normal cell lines through percentage cell viability at different concentrations of extracts. The results were expressed by mean value (n = 3) of % age cell viability. The lower value of cell viability, the higher the inhibition of cancerous cells. The results were compared by two standard anticancer drugs such as doxorubicin and cyclophosphamide [22]. This study observed the characteristic cytotoxic effect of *P. cornuta* extracts in A549 and MDA-MB-231 cell lines. However, at lower concentrations, the effect of *P. cornuta* extracts is not statistically significant, particularly PCB and PCN extracts against Caco-2 cells, all PC extracts against HepG2 cells and, PCB, PCN extracts in NCI-H1347, which points to possible future research on the protective effect of extracts towards normal cells at a concentration less than 1 µg/mL.

In addition, extracts in different solvents showed a slightly different inhibition pattern against a specific type of cancerous cell lines due to differences in their polarities [23]. Chloroform extracts of the *P. cornuta* plant altogether showed the highest cytotoxic effect in Caco-2, A549, and MDA-MB-231 cancerous cells also signify the antibacterial activity results. The moderate anti-proliferative effect of *P. cornuta* extracts in hepG2 cells may demand a higher dose to produce effective inhibition, such as the significant inhibitory effect of *P. spinosa.* L. flower extracts in HepG2 cells were 150–200 µg/mL [24]. In most *Prunus* species, the cytotoxicity potential was attributed to glycosides (cyanogenic) as reflected by the preliminary chemical characterization in Table 2 and the existence of quercetins in the plant extracts. The cytotoxic effect of the QS plant has been attributed to the presence of tannins, terpenoids, flavonoids, and glycosides. This correlation of pharmacological effect and chemical composition has also been reported in various *Quercus* species [15,19].

## 4. Materials and Methods

### 4.1. Plant Samples Preparation

With the help of local inhabitants, fresh plant material of *Prunus cornuta* and *Quercus semicarpifolia,* locally known as Bhareet and Banjar plants, were collected during the spring season from Pallas valley, District Kohistan, Khyber Pakhtunkhwa province, located at 36.6° N, 73.00° E, 315 km from capital city Islamabad as shown in Figure 1. Biological authentication was performed by consulting plant taxonomists at the herbarium, Plant Sciences department, Quaid e Azam University, Islamabad. After collection, plant material was subjected to the removal of dust particles by brushing off extra debris and washing with distilled water. The washed plant material was shade-dried. During drying, plant material was checked regularly for fungal infection or chances of any other contamination. Plants were then crushed and ground to a fine powder using a grinding mill. To avoid mixing one plant material with another, each time, the grinding mill was cleaned, washed with 70% ethanol, and dried properly. The powdered plant material was stored in resealable zipper bags, appropriately labeled, and kept in a dark and cool place (below 10 °C).

Extracts were prepared by the maceration method. Dried leaves and branches were pulverized mechanically through 60 mesh willy mill and soaked into solvents of different polarities (20 g/200 mL) in conical flasks (250 mL), covered with cotton and aluminum foil. The material was placed on a shaker for 72 h. After that, we filtered the residue through Whatman No. 1 filter paper, and the filtrate was evaporated at room temperature under shade. The concentrated extracts were stored at 4 °C until further use [25,26].

### 4.2. Phytochemical Screening

The methanolic extracts of selected plants were subjected to the preliminary screening of phytochemicals such as alkaloids, flavonoids, saponins, tannins, and carbohydrates [14,27,28,29,30].

#### 4.2.1. Test for Alkaloids

##### Mayer’s Reagent Test

For identifying alkaloids, 2 mL of conc. hydrochloric acid (HCl) was added to 2 mL of plant extract, followed by the drop-by-drop addition of Mayer’s reagent. The formation of white precipitate or green color appearance revealed the presence of alkaloids [29].

##### Hager’s Test

A few drops of saturated picric acid (Hager’s reagent) were added to the 2 mL plant test extract. The appearance of bright yellow precipitates revealed the existence of alkaloids in test samples [27,28,29].

#### 4.2.2. Test for Saponins

This test was performed by adding 2 mL distilled water to 2 mL plant extract and shaking vigorously for 5 min in a test tube. The formation of a foam layer (1 cm) indicated the presence of saponins [27,28].

#### 4.2.3. Test for Flavonoids

A few drops of FeCl_3_ solution were added to 1 mL of plant extract samples. Flavonoid presence results in blackish-red precipitation [29,30].

#### 4.2.4. Test for Tannins

##### Alkaline Reagent Test

Plant extracts (2 mL) were added to 1N NaOH (2 mL) and mixed thoroughly. The appearance of yellow to red precipitates confirmed the presence of tannins [29,30].

##### Ferric Chloride Test

A volume of 2 mL of 5% FeCl_3_ was added to 1 mL of plant extract samples. The appearance of dark blue or greenish-black color indicated the presence of tannins in test samples [29,30].

#### 4.2.5. Test for Glycosides

A Keller Killiani test was performed for glycosides detection. A volume of 1 mL of glacial acetic acid was added to 1 mL of plant extract samples, followed by cooling, and adding two drops of FeCl_3_. After that, we carefully added 0.5 mL of H_2_SO_4_ along the sides of the test tube. A reddish-brown ring at the junction of two layers indicated glycoside existence [29,30].

#### 4.2.6. Test for Sterols

Salkowski test: 5 mL of chloroform and 2 mL of plant extract samples were mixed in this test. This was followed by careful addition of 1 mL of conc. H_2_SO_4_ along the walls of the test tube. Reddish-brown color in the lower layer indicated sterols presence in the test sample [27,28,29,30].

#### 4.2.7. Test for Phenols

##### Ellagic Test

A few 5% glacial acetic acid drops were added to 1 mL of plant extract samples. Then a few drops of 5% NaNO_2_ were added. Muddy brown color revealed the existence of phenols [29,30].

#### 4.2.8. Test for Carbohydrates

Benedict’s test was used for carbohydrate detection. A few drops of benedict’s reagent/alkaline solution of cupric citrate complex were mixed with test samples followed by boiling in the water bath. Reddish-brown precipitate indicated the presence of carbohydrates in the test substance [29].

#### 4.2.9. Test for Proteins

##### Xanthoproteic Test

A volume of 1 mL of plant extracts was subjected to a few drops of concentrated nitric acid. Yellow color formation revealed the presence of proteins in test samples [28,29,30].

#### 4.2.10. Test for Anthraquinones

A few drops of 2% HCl were added to the plant extract solution. The formation of red precipitation indicated anthraquinones in the test substance [29]. Next, 2 mL of extract was mixed with 2 mL of benzene solution followed by 1 mL of 10% ammonia solution. The appearance of red coloration indicated the presence of anthraquinones in the plant extract [30].

#### 4.2.11. Test for Phlobatanins

A few 10% ammonia solution drops were added to 1 mL of plant extract sample. Pink color precipitation signified the existence of Phlobatanins in test samples [29,30].

### 4.3. Antimicrobial Assay

#### 4.3.1. Antibacterial Activity

##### Bacterial Strains

Antibacterial activity was performed against the following strains: *Klebsiella pneumoniae* (82,431), *Escherichia coli* (52,321), *Bacillus subtilis*, *Salmonella enterica*, and *Acinetobacter baumannii*.

##### Agar-Well Diffusion Method

The method of Boyanova et al. was used to prepare microbial cultures [31]. They were grown in sterile Muller Hinton Agar (MHA) using sterile Petri dishes. Microbial cultures with different strains (100 mL) were added to MHA (100 mL) and poured into specified Petri dishes. Wells for both samples and controls were made into each agar plate using a sterile cork borer. Then 40 mg of plant extract were dissolved in DMSO (4%; 1 mL), and 100 µL of the sample was transferred into the 6 mm wells. The plates were then incubated for 48 h at 37 °C. Finally, the zone of inhibition was measured using a ruler (*n* = 3). Kinamycin was used as a positive control.

#### 4.3.2. Antifungal Activity

##### Fungal Strains

Antifungal activity was evaluated against the following fungal strains: *Rhizopus oryzae*, *Aspergillus flavus* (FCBP 0064), *Aspergillus niger* (FCBP 0198), and *Pythium* species.

##### Agar-Well Diffusion Method

The agar-well diffusion method was used for antifungal activity. The sample material was prepared by dissolving 40 mg of each text extracts into 1 mL of DMSO stock solution. Two milligrams of terbinafine were mixed with 1 mL DMSO solvent for the positive control. The negative control was pure DMSO solvent. The mycelial inhibition of fungal strains was performed by pouring and solidifying 25 mL of SDA into Petri plates. The refreshed fungal strains were streaked on the media surface with the help of sterile cotton buds. Wells were made in each of the Petri plates using a sterile cork borer. About 100 µL of stock solution of plant extracts was added into wells and allowed to diffuse for 2 h. Then we sealed the plates with parafilm and placed them in the upside-down position for 48 h at 28 °C in the incubator. The diameter of the zone of inhibition was measured, which was a cleared zone around the well. The experiment was performed in triplicates. The reading measurement was done in different directions, and the average value was taken.

##### Agar Slanting Method

The percentage of antifungal activity of crude extracts was evaluated against the following fungal pathogens, i.e., *R. oryzae*, *Fusarium fujikuroi,* and *Pythium* sp. The studied fungal strains were cultured on Sabouraud dextrose agar (SDA) and incubated at 37 °C for 24 h. The inoculum was poured into sterilized test tubes containing 4 mL of SDA. In each test tube having SDA, approximately 65 µL of the test sample (40 mg/mL, DMSO) was added. The media was solidified and incubated at 37 °C for 3–7 days. Terbinafine (2 mg/mL) and DMSO were used as positive and negative controls, respectively [32]. After incubation, the linear growth inhibition was determined, and percent inhibition was calculated using Equation (1).

(1) $$\%\;\mathrm{inhibition}\;=100-\frac{\mathrm{Linear}\;\mathrm{growth}\;\left(\mathrm{test}\right)}{\mathrm{Linear}\;\mathrm{growth}\;\left(\mathrm{control}\right)}\times 100$$

### 4.4. Anticancer and Safety Activities

#### 4.4.1. Cancer Cell Lines and Primary Human Cell Culture

Human hepatocellular carcinoma cell line HepG2, human intestinal epithelial cell line Caco-2, human lung adenocarcinoma cell line A549, human breast adenocarcinoma cell line MDA-MB-231, and human non-small cell lung cancer cell line NCI-H1437 (Korea cell line bank, Seoul, Korea) were cultured in RPMI-1640 media supplemented with 10% FBS and 1% (*v/v*) penicillin and streptomycin solution. Cultured cells were kept in a humid atmosphere at 37 °C with 5% CO_2_. After 80 to 90% confluency, the cells were expanded up to 3 passages before seeding and washed with Dulbecco’s phosphate buffer saline (DPBS) (Cat# 14190144, ThermoFisher, Waltham, MA, USA) to remove cell debris and metabolites before adding fresh media. Cells at 90% confluency were trypsinized with 0.05% Trypsin-EDTA solution (Cat# 25300054, Thermo Fisher, USA), then suspended in freshly prepared RPMI-1640 media containing a specified concentration of FBS [22,33,34,35]. Human primary pulmonary alveolar epithelial cells (HPAEpiC) (Science Cell Research laboratories, Carlsbad, CA, USA) were revived according to the manufacturer’s instructions. A T-25 flask was coated with Poly-L-lysine (Sigma Aldrich) at a concentration of 2 µg/cm^3^ kept at 37 °C for 24 h. The flask was rinsed before adding culture medium containing alveolar epithelial cells (AEpiCM, cat#3201), minimal eagle medium, epithelial growth supplement (5 mL), 10% FBS, 1% penicillin-streptomycin solution with 5% carbon dioxide at 37 °C. In addition, human primary renal proximal epithelial cells (RPTEC) were cultured in Epithelial Cell Medium (EpiCM, cat#4101).

#### 4.4.2. MTS Based Cell Viability Assay Procedure

MTS assay was used to determine the number of viable cells. The cells were seeded in 96-well microplates at a concentration of 5000 cells per well. After a 24 h incubation, the cells were subjected to various concentrations (from 1 ng/mL to 100 µg/mL) of 100 µL of serially diluted plant extracts prepared in solvents of different polarity. The cells containing media only for blank value and the anticancer drug was also incorporated for positive control into wells. In addition, the DMSO alone was added to another set of cells for solvent control. After 48 h of incubation, about 25 µL of MTS reagent (ThermoFisher, Waltham, MA, USA) was added, and the cells were allowed to incubate at 5% CO_2_ and 37 °C for 30 min. The absorbance of cells was measured at 490 nm by a multimode microplate reader (SpectraMax iD3, Molecular Devices, Xuhui, Shanghai, China). Percentage cell viability was calculated by using Equation (2) [36,37].

(2) $$\%\;\mathrm{age}\;\mathrm{cell}\;\mathrm{viability}\;=\frac{\mathrm{Control}-\mathrm{Blank}}{\mathrm{Sample}-\mathrm{Blank}}\times 100$$

### 4.5. Statistical Analysis

Statistical analysis was conducted using one-way ANOVA and Dunnett’s post hoc test (GraphPad Software Inc., San Diego, CA, USA); data were presented as mean ± SEM. All the tests were performed in triplicate. The values of *p* < 0.001 were considered statistically significant.

## 5. Conclusions

The present study concludes that the extracts of *Prunus cornuta* and *Quercus semicarpifolia* exhibited potent bioactivity against bacterial strains and human carcinoma cells with a minimal toxic effect on healthy cell lines. The *P. cornuta* extract significantly inhibited the *A. buamanii* and *S. enterica* strains and induced characteristic cell death in breast (MDA-MB321) and lung (A549) cell lines. The methanolic extracts of *Q. semicarpifolia* showed potent antibacterial activity against *A. baumannii* and *E. coli*. Additionally, *Q. semicarpifolia* extracts showed considerable cytotoxic effect against breast (MDA-MB231) and gut (Caco-2) cancer cells. The effective bioactive potential of both plants provides the basis for a new natural source on the drug ability list of pharmacognosy platforms.

## Figures and Tables

**Table 1:** Qualitative phytochemical analysis of methanolic crude extracts of selected plants.

Constituents	Tests	PCM	QSM
Alkaloids	Mayer’s test	+	+
	Hager’s test	+	+
Tannins	FCl_3_ test	+	+
	Alkaline reagent test	+	+
Saponins	Foam test	+	+
Flavonoids		+	+
Glycosides		+	+
Sterols		N	+
Phenols		N	+
Carbohydrates		N	N
Anthraquinones		+	N
Phlobatanins		−	−
Anthocyanin		−	−
Quinones		+	+
Protein	Xanthoproteic test	N	

Illustrated the qualitative indication of phytochemicals present in plant methanolic extracts. Abbreviations: + sign, present; − sign, absence; N, Not indicated.

**Table 2:** Antibacterial activity of *P. cornuta* and *Q. semicarpifolia* extracts.

Extract Solvents 4000 µg/mL	*B. subtilis*	*E. coli*	*K. pneumoniae*	*S. enterica*	*A. baumannii*
	Zone of Inhibition (mm)				
PCB	11.5	11.0	11.5	14.5	16
PCC	13	13	12	13	14
PCE	12	11.5	13	13	13
PCM	11	11	12	14	13
PCN	12	14	11	15.5	15
QSB	12	13	12	8	15
QSC	12.5	11	13	8	14
QSE	12	12.5	11	10	16
QSM	14	15	13	10	18
QSN	11	12.5	12.5	7	16
P	12	13	16	15	12
N	-	-	-	-	-

Values are means of triplicate (*n* = 3), - means no activity, extracts in butanol (PCB, QSB), chloroform (PCC, QSC), ethyl acetate (PCE, QSE), methanol (PCM, QSM), and *n*-hexane (PCN, QSN). Low activity (7–10 mm); moderate (11–13 mm); high activity (14–18 mm).

**Table 3:** Antifungal activity of *P. cornuta* and *Q. semicarpifolia* extracts.

Extract	*R. oryzae*	*A. flavus*	*A. niger*	*Pythium* sp.
	Zone of Inhibition (mm)			
PCB	-	-	-	-
PCC	-	-	-	-
PCM	16.5	-	-	1.5
PCN	16	-	-	-
QSB	16	-	-	-
QSC	16	-	-	-
QSE	21	-	-	-
QSM	16	-	-	2.25
QSN	16.5	-	-	-
DMSO	-	-	-	-
Terbinafine	30	35	32.5	36

Values are means of triplicate (*n* = 3), - means no activity. Extracts in butanol (PCB, QSB), chloroform (PCC, QSC), ethyl acetate (PCE, QSE), Methanol (PCM, QSM), and *n*-hexane (PCN, QSN).

**Table 4:** Percentage inhibition of mycelial growth of *F. fujikuroi, R. oryzae,* and *P. ultimum* by plant extracts.

Extracts	Fungal Isolates		
	*F. fujikuroi*	*R. oryzae*	*P. ultimum*
PCB	*54*	*62*	*38*
PCC	59	59	39
PCE	52	64	40
PCM	55	67	43
PCN	50	60	44
QSB	49	57	-
QSC	54	53	-
QSE	46	54	-
QSM	44	57	-
QSN	37	48	-
Positive control/Terbinafine	56	79	62
Negative control	-	-	-

%Age mycelial inhibition expressed as mean ± SD (*n* = 3). Lower inhibition = 20–30%, moderate inhibition = 40–50%, high inhibition = 60–80%. Positive control: Terbinafine.

## Data Availability

The data supporting this study are available from the corresponding author upon reasonable request.

## Supplementary Materials

The following are available online at https://www.mdpi.com/article/10.3390/plants11010048/s1, Figure S1: Antibacterial activity *Prunus cornuta* extracts against infectious bacterial species, Figure S2: Fungal inhibition on SDA media by plant extracts in butanol, chloroform, ethyl acetate, methanol, and n-hexane extracts. *Pythium* sp.*1, A niger, A. Flavous* sp. exhibited no significant inhibition except *Royzae* sp. with average 15% inhibition by butanol, chloroform, ethyl acetate, methanol, and 0.0% by n-hexane extracts, Figure S3: Antibacterial activity *Quercus semicarpifolia* extracts against infectious bacterial species.
