# Supplementary material for: Evaluation of Antimicrobial and Anticancer Activities of Selected Medicinal Plants of Himalayas, Pakistan
Source: Plants (Basel). 2021 Dec 24;11(1):48. doi: 10.3390/plants11010048 (PMC8747275; doi:10.3390/plants11010048)
Supplement: Supplementary file 1 [file plants-11-00048-s001.zip › plants-1512470-supplementary.pdf]

Supplementary Data:

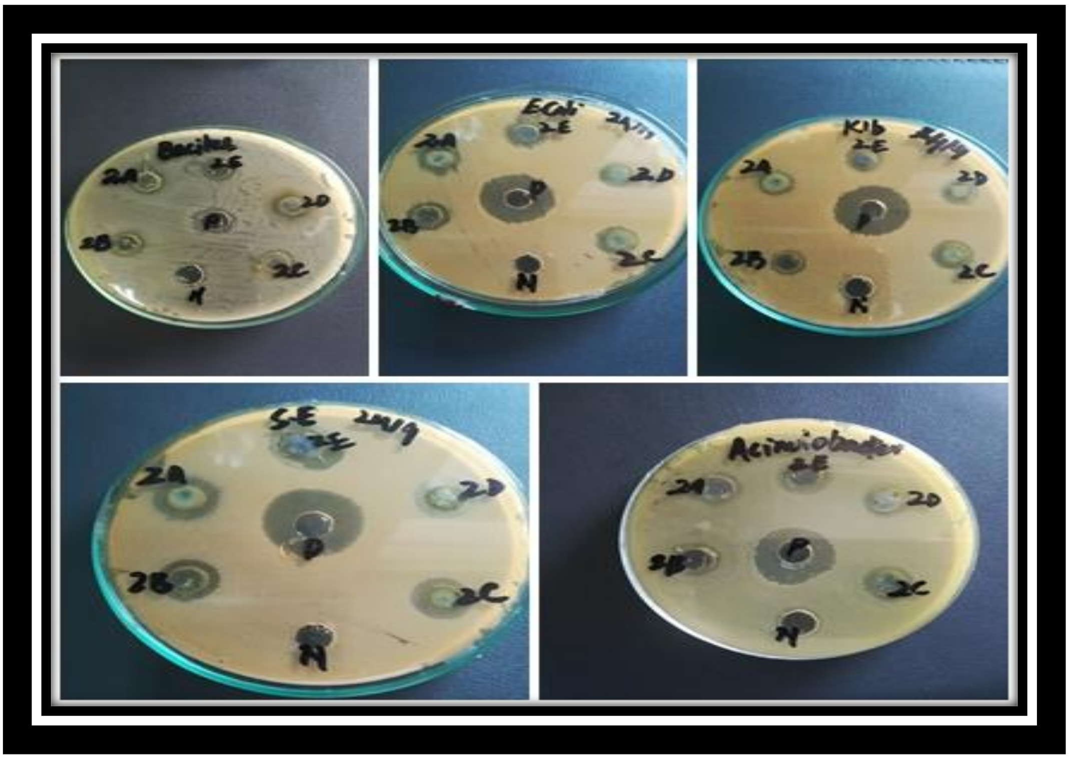

Figure S1. Antibacterial activity *Prunus cornuta* extracts against infectious bacterial species.

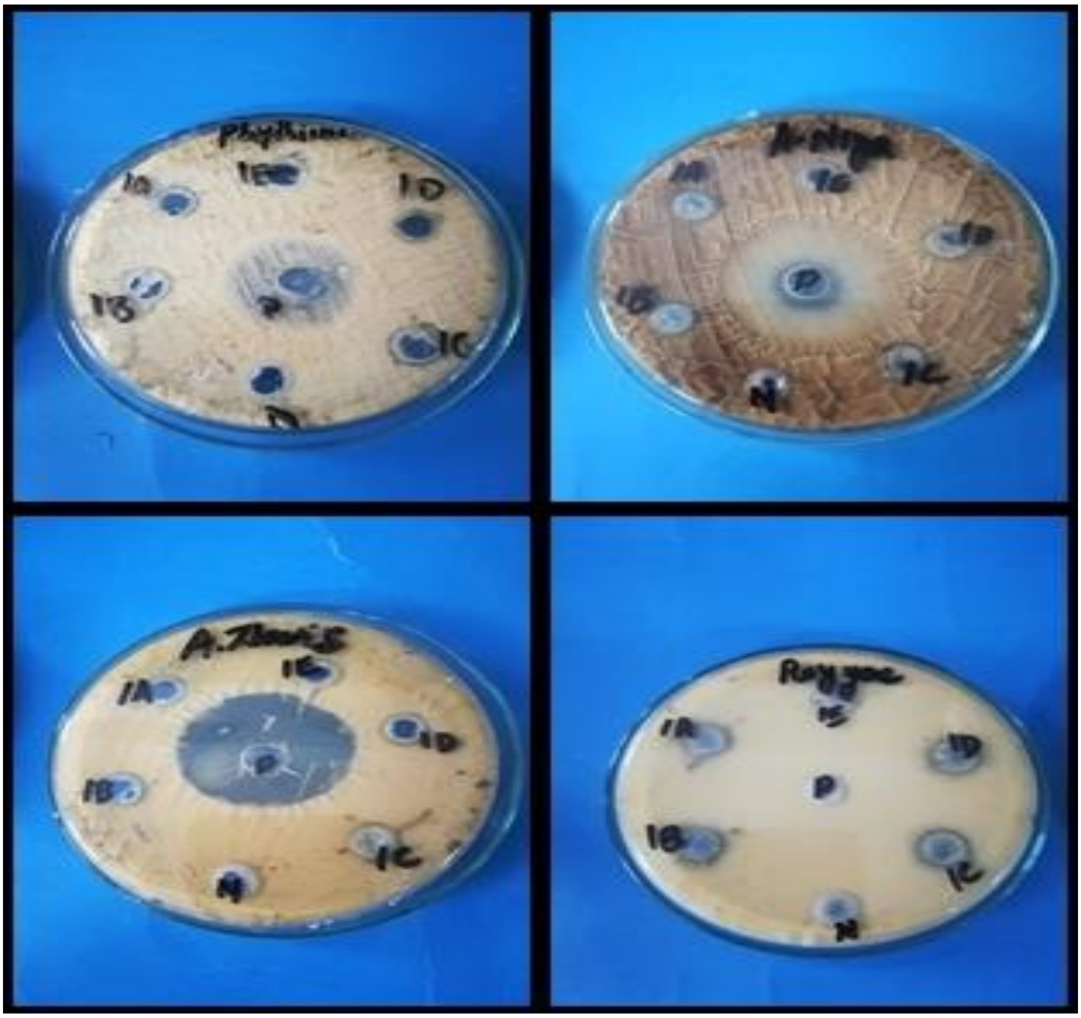

Figure S2. Fungal inhibition on SDA media by plant extracts in butanol, chloroform, ethyl acetate, methanol, and n-hexane extracts. *Pythium sp.1, A niger, A. Flavous sp*. exhibited no significant inhibition except *Royzae sp*. with average 15 % inhibition by butanol, chloroform, ethyl acetate, methanol, and 0.0 % by n-hexane extracts.

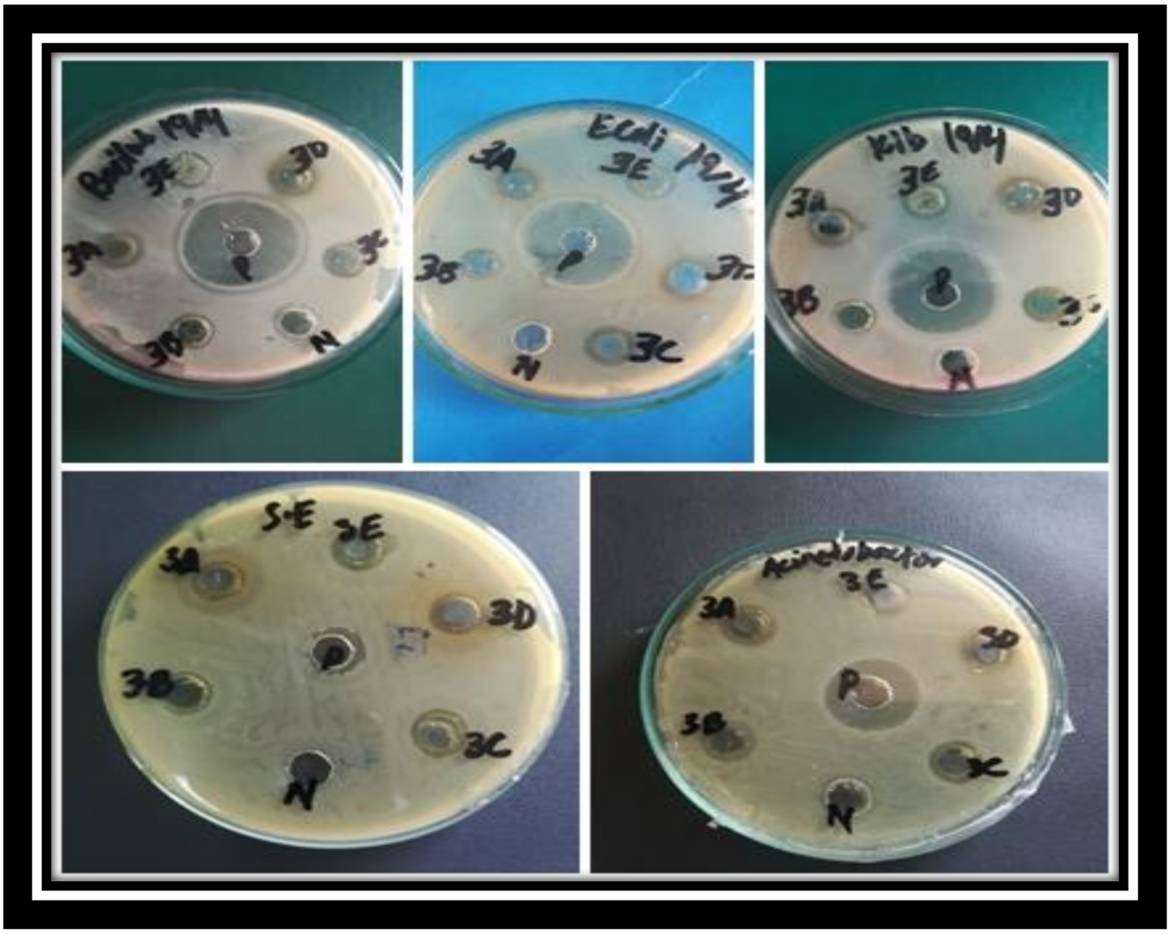

Figure S3. Antibacterial activity *Quercus semicarpifolia* extracts against infectious bacterial species.
